# Grass carp Trim47 restricts GCRV infection via SPRY domain-mediated autophagic degradation of nonstructural proteins and disruption of viral inclusion bodies

**DOI:** 10.3389/fimmu.2025.1623014

**Published:** 2025-07-10

**Authors:** Wei Yan, Yang Chen, Dong Yan, Jie Zhang, Ming Xian Chang

**Affiliations:** ^1^ State Key Laboratory of Breeding Biotechnology and Sustainable Aquaculture (CAS), State Key Laboratory of Freshwater Ecology and Biotechnology, Institute of Hydrobiology, Chinese Academy of Sciences, Wuhan, Hubei, China; ^2^ College of Advanced Agricultural Sciences, University of Chinese Academy of Sciences, Beijing, China

**Keywords:** grass carp Trim47, grass carp reovirus, autophagic degradation, gcTRIM47-PYD1 recombinant *Saccharomyces cerevisiae* biologics, SPRY domain

## Abstract

Trim47, a TRIM C-VII subgroup protein characterized by a conserved SPRY domain, has been primarily studied for its ubiquitin-dependent roles in mammals. This study reports a paradigm-shifting finding in teleost immunology: grass carp Trim47 (gcTrim47) employs its SPRY domain to execute a novel, ubiquitin-independent antiviral pathway, selectively degrading GCRV-I nonstructural proteins NS38/NS80 via autophagy-mediated clearance. Unlike mammalian TRIMs, gcTrim47 antiviral activity is strictly dependent on its SPRY domain—devoid of RING/B-box domains critical for E3 ligase function—revealing an evolutionarily divergent mechanism where substrate-targeting specificity, not ubiquitination, drives viral replication factory (viral inclusion body, VIB) dismantling. Functional assays demonstrated that gcTrim47 overexpression in CIK cells reduced viral titers and suppressed VIB formation, with SPRY domain deletion ablating these effects. *In vivo*, a yeast surface-display platform presenting gcTrim47-PYD1 conferred 32.94% relative percent survival (RPS) against GCRV-II infection, the first reported use of a TRIM family protein as an antiviral immunogen in grass carp. This strategy mitigated splenic/kidney viral loads and alleviated histopathological damage, including tubular necrosis and inflammatory infiltration. The successful application of this mechanism into a yeast-based immunization strategy highlights its potential for developing novel antiviral biotherapeutics in aquaculture.

## Highlights

- Overexpression of gcTrim47 inhibits GCRV-I infection
- gcTrim47 degrades the nonstructural proteins NS38 and NS80 via the SPRY domain
- gcTrim47 degrades the nonstructural proteins NS38 and NS80 via the autophagy pathway
- gcTrim47 impairs the production of VIBs and GCRV-I replication via the SPRY domain
- Grass carp immunized with gcTrim47-expressing yeast confers protection against GCRV-II infection

## Introduction

Grass carp (*Ctenopharyngodon idella*) represents a pivotal freshwater aquaculture species in China, contributing significantly to global fish production and rural economies. However, its industry faces severe challenges from Grass Carp Hemorrhagic Disease (GCHD), caused by Grass Carp Reovirus (GCRV), which imposes substantial economic losses due to high juvenile mortality. As a member of the *Reoviridae* family and *Aquareovirus* genus, GCRV is an icosahedral virus with a 70–80 nm diameter, featuring an 11-segmented double-stranded RNA genome (designated S1 to S11) (1). This genome encodes seven structural proteins (VP1–VP7) and five non-structural proteins (NS16–NS88), with NS80 and NS38 being critical for forming viral inclusion bodies (VIBs). These VIBs serve as specialized hubs for recruiting viral and host factors, optimizing replication and assembly processes (2–4). Genetic analysis of the VP6 gene defines three GCRV genotypes (I, II, III), with GCRV-II distinguished by its extreme virulence, inducing mortality rates of 80–90% in juvenile populations (5). While GCRV-I and GCRV-II share conserved core replication machinery and genomic segmentation, they diverge significantly in structural protein-coding regions (e.g., VP7, VP5, VP4) and virulence-associated factors (6). These differences underlie distinct host interaction patterns and pathogenic outcomes, highlighting the need for genotype-specific insights into virus-host dynamics.

GCRV nonstructural proteins serve as critical effector molecules in orchestrating immune evasion strategies. Our laboratory has previously demonstrated that during GCRV-I infection, the virus exploits grass carp vitamin D receptors (VDRs)-host factors that interact with viral nonstructural proteins to facilitate the production of viral inclusion bodies (VIBs) (7). Additionally, we identified grass carp oxysterol-binding protein 1 (gcOSBP1)-a host factor mediating cholesterol accumulation to enhance GCRV replication-as an interacting partner for the essential VIBs components NS38 and NS80. Recruitment of gcOSBP1 by these viral proteins is critical for facilitating VIBs formation during infection (8). Beyond hijacking host factors to promote VIBs formation, GCRV employs sophisticated mechanisms to subvert the type I interferon (IFN) response. Recent investigations reveal that NS38 or NS80 of GCRV interact with specific components of the RIG-I-like receptor (RLR) antiviral signaling pathway, then sequester these signaling molecules within cytoplasmic VIBs. This spatial isolation disrupts the formation of functional signaling complexes, effectively dampening RLR-mediated antiviral immunity and type I IFN response (9). GCRV also utilizes grass carp liver X receptor α (gcLXRα) to competitively bind the transcriptional coactivator CREB-binding protein (CBP) away from IFN regulatory factor 3 (IRF3), thereby antagonizing the host’s type I IFN response at the transcriptional level (10).

The tripartite motif (TRIM) protein family, encompassing over 70 members across nearly all multicellular taxa, serves as a critical nexus of immune regulation and E3 ubiquitin ligase activity in innate immunity (11). The typical structures of TRIM proteins include the N-terminal RING domain (mediating ubiquitination), the B-box domain (involved in protein-protein interactions), and the coiled-coil domain (mediating oligomerization). Many TRIM family members also possess divergent C-terminal regions, such as the PRY/SPRY domain for substrate recognition (12). Functionally, TRIM proteins act as key modulators of host antiviral innate immunity through multiple interconnected mechanisms. Firstly, they regulate pattern recognition receptor (PRR) signaling pathways, such as those mediated by Toll-like receptors (TLRs) and RIG-I-like receptors (RLRs), to fine-tune downstream IFN responses (13). Secondly, they orchestrate autophagic processes and STING or NF-κB signaling pathway, integrating cellular stress with antiviral defense (14). Thirdly, certain TRIMs exhibit direct antiviral activity by targeting viral components for ubiquitination and degradation, as exemplified by TRIM5α’s restriction of retroviral capsids and Trim2b’s restriction of spring viremia of carp virus (SVCV) via autophagy lysosomal pathway (14–17).

Teleost fish TRIM proteins, key E3 ubiquitin ligases in antiviral immunity, exhibit remarkable functional diversification and species-specific adaptations (18). Studies highlight their roles in modulating IFN signaling via ubiquitination of IRF3 or interaction with TBK1/TRAFs to combat viruses like SVCV, iridovirus and nodavirus (19, 20). Teleost-specific subfamilies like finTRIMs have evolved through genome duplication, acquiring novel domains (e.g., SPRY-PRY) and RNA-binding activity to fine-tune IFN response (21, 22). For instance, the crucian carp finTRIM FTRCA1 acts as both an E3 ligase and RNA-binding protein, degrading TBK1 via autophagy-lysosomal pathways while targeting STING1/IRF7 mRNAs for degradation through the RNA-induced silencing complex (RISC) complex (22, 23). Conversely, zebrafish ftr83 enhances IFN signaling through its RING/B30.2 domains, providing antiviral protection against RNA viruses (24). These findings underscore the multifaceted nature of fish TRIM proteins, which integrate ubiquitination, E3 ligase activity, and RNA binding to orchestrate species-specific IFN pathway modulation.

Trim47, a member of the TRIM family subgroup C-VII, harbors a C-terminal SPRY (SPla and RYanodine receptor) domain and exhibits high sequence homology with the well-characterized mammalian TRIM25. In mammals, Trim47 functions as a multifunctional regulator, driving tumor progression through ubiquitin-dependent proteolysis and oncogenic signaling while participating in inflammatory and immune pathways, positioning it as a therapeutic target in cancer (25–27). Cross-species studies have revealed its immunomodulatory roles: genetic deletion of Trim47 attenuates NF-κB/MAPK-mediated inflammation in murine acute lung injury models (28), while in zebrafish, Trim47 deficiency impairs complement activation and innate immune surveillance, increasing susceptibility to SVCV infection (29). In teleosts, overexpression of common carp TRIM47 in FHM cells reduces SVCV-G gene expression, hinting at conserved antiviral potential. Despite these insights, the immune regulatory roles of Trim47 in grass carp—a critical aquaculture species—remain uncharacterized, particularly in the context of its highly pathogenic virus, GCRV. How Trim47 interacts with GCRV nonstructural proteins (e.g., NS80, NS38), which are essential for VIBs formation and immune evasion, is entirely unknown. The functional significance of the SPRY domain in mediating host-virus interactions and autophagic degradation pathways in fish TRIMs has not been elucidated. Given the extreme virulence of GCRV-II, whether Trim47 exhibits conserved activity against divergent GCRV genotypes (I/II) remains unaddressed. This research fills these gaps by demonstrating that grass carp Trim47 (gcTrim47) employs its SPRY domain to induce selective autophagic degradation of GCRV NS80/NS38, dismantling viral replication factories. Additionally, the development of a yeast surface display system validates gcTrim47’s *in vivo* antiviral efficacy against GCRV-II, establishing validation of the concept for TRIM-based biotherapeutics in aquaculture. These findings resolve fundamental knowledge gaps in fish TRIM biology and offer translational strategies to combat GCRV, a critical threat to global freshwater aquaculture.

## Materials and methods

### Cells, virus, and plasmids

*Ctenopharyngodon idella* kidney (CIK) cells were routinely maintained in Minimum Essential Medium (MEM, Gibco) supplemented with 10% heat-inactivated fetal bovine serum (FBS, Gibco), 100 U/mL penicillin, and 100 μg/mL streptomycin at 28°C in a 5% CO_2_ incubator. Grass carp reovirus stain (GCRV-I, GCRV-873) was propagated in CIK cells using MEM containing 2% FBS. Grass carp reovirus type II (GCRV-II, GCRV-GD108) was propagated in grass carp by intraperitoneal injection (30). Plasmid constructs included commercially available vectors: pTurboGFP (Evrogen), p3×FLAG-CMV-14 (Sigma-Aldrich), and laboratory-generated plasmids such as PCI-neo (constitutive neomycin resistance), YFP-FLAG, NS80-GFP (GCRV NS80 protein fused to green fluorescent protein), and PCI-neo-NS38 (GCRV NS38 expression vector) (9). Domain-deletion or truncated mutants of gcTrim47, including gcTrim47-ΔRING (RING domain deleted), gcTrim47-ΔBbox (B-box domain deleted), gcTrim47-ΔSPRY (SPRY domain deleted), and gcTrim47-SPRY (only containing SPRY domain), were generated by PCR amplification using Phusion High-Fidelity DNA Polymerase (NEB). Specifically, gene-specific primer pairs (**Table 1**) were designed to amplify full-length gcTrim47 and its truncated variants, incorporating HindIII and BamHI restriction sites at the 5’ and 3’ ends, respectively. Amplicons were double-digested with these enzymes and ligated into the linearized p3×FLAG-CMV-14 vector using T4 DNA ligase (NEB). The gcTrim47-PYD1 recombinant plasmid was constructed by PCR amplifying the target coding sequence with PYD1 vector-specific primers (Invitrogen) and inserting it into the yeast surface display backbone via homologous recombination. All plasmid constructs were validated by Sanger sequencing to confirm correct open reading frames and absence of mutations. Primer sequences are provided in **Table 1**.

**Table 1:** Primers used for the present study.

Primers	Sequence (5’ to 3’)	Application
TRIM47-F	CCAAGCTTATGGCCACTGCCGGAGAT	Ligated to p3xFLAG-CMV™-14 vector
TRIM47-R	CGGGATCCGTAGAAGTGACATATTTGTAGTCGGCTAC	
Trim47-ΔRING-F	CCAAGCTTATGGGGAAATGTGAGCGACACCACG	
Trim47-ΔRING-R	CGGGATCCACATATTTGTAGTCGGCTACCCGGGT	
Trim47-ΔBbox-F	CCCGTGAAAATGGACCCAGAGGTTGTT	
Trim47-ΔBbox-R	AACAACCTCTGGGTCCATTTTCACGGG	
Trim47-ΔSPRY-F	CCAAGCTTATGGCCACTGCCGGAGATTCG	
Trim47-ΔSPRY-R	CGGGATCCTACAGGAGCAACATCATGGTGTCTGTG	
Trim47-SPRY-F	CCCAAGCTTATGTTGACTCTAGATTTAGACACCG	
Trim47-SPRY-R	CGGGATCCACATATTTGTAGTCGGCTACCC	
TRIM47-PYD1-F	CGGGATCCATGGCCACTGCCGGAGAT	Ligated to PYD1 vector
TRIM47-PYD1-R	CGGAATTCGTAGAAGTGACATATTTGTAGTCGGCT	
gcLC3-F	CGGAATTCATGCCTTCGGAAAAGACATTTAAAC	Ligated to GFP vector
gcLC3-R	CGGGATCCAACTGAGGACACGCAGTTCC	
GCRV-II-S6-F	AGCGCAGCAGGCAATTACTATCT	qRT-PCR
GCRV-II-S6-R	ATCTGCTGGTAATGCGGAACG	
TRIM47-F	CAGTATTCCAGCGGTCAG	
TRIM47-R	AGACACAGGCTCCAGTAG	

### Experimental fish

Healthy grass carps (mean weight 10 ± 1 g) were obtained according to our previous report (30). Fish were acclimatized in aerated freshwater with temperature maintained at 25 ± 2°C for two weeks, and fed with a commercial pelleted diet at 3% body weight per day through-out the study. All animal experiments were conducted in accordance with the Guiding Principles for the Care and Use of Laboratory Animals and were approved by the Institute of Hydrobiology, Chinese Academy of Sciences (IHB/LL/2022048).

### Abs and reagents

The anti-FLAG mouse mAb (#F3165), anti-HA rabbit mAb (#51064-2-AP) and anti-p62 (P0067) were purchased from Sigma-Aldrich. The anti-GAPDH mouse mAb (#60004-1-Ig) was purchased from Proteintech. The anti-NS38, anti-NS80, anti-VP3 and anti-VP5 polyclonal rabbit Abs against the GCRV-873 strain were prepared previously (31). The anti-V5 mouse monoclonal antibody, goat anti-mouse Ig-HRP conjugate secondary Ab, goat anti-rabbit IgHRP conjugate secondary Ab, Alexa Fluor 594 conjugated secondary Ab against rabbit IgG, Alexa Fluor 488 conjugated secondary Ab against mouse IgG, DAPI, RevertAid First Stand cDNA Synthesis Kit (#K1622), Lipofectamine 3000, and protease inhibitor mixture were purchased from Thermo Fisher Scientific. The FLAG Immunoprecipitation Kit was purchased from Sigma-Aldrich. Trizol reagent (#15596026) was purchased from Invitrogen. MG132 (S2619), 3-methyladenine (3-MA; S2767), and ammonium chloride (NH_4_Cl; E0151), were purchased from Selleck Chemicals.

### Viral infection assays

To investigate the roles of gcTrim47, gcTrim47-ΔRING, gcTrim47-ΔBbox, and gcTrim47-ΔSPRY during GCRV infection, CIK cells (1×10 cells/well) in 24-well plates were transfected with 500 ng of YFP-FLAG, gcTrim47-FLAG, gcTrim47-ΔRING-FLAG, gcTrim47-ΔBbox-FLAG, or gcTrim47-ΔSPRY-FLAG plasmids. At 24 hours post-transfection, cells were either infected with GCRV at a multiplicity of infection (MOI) of 1 or left uninfected. Supernatants were collected at 24 hours post-infection (hpi) for viral titer determination using the standard TCID_50_ assay. Subsequently, cells were fixed with 4% paraformaldehyde for 2 hours, stained overnight with 0.05% crystal violet solution, carefully rinsed with tap water, air-dried, and photographed for documentation.

### Immunofluorescence assays

To determine the possible colocalization of gcTrim47 and its domain mutants with NS38 or NS80 protein, CIK cells (24-well plates, 2.5 × 10^5^ per well) were transfected with 500 ng indicated plasmid for 36 h, and then exposed to GCRV-I (MOI = 1) for 24 h or left untreated. The cells were washed with PBS to remove non-adherent virions, and the infected cells were maintained in 2% FBS MEM. At 18 hpi, the cells were treated with 10 mM 3-MA at 28°C for 6 h or left untreated. Then, the cells were washed twice with PBS and fixed with 4% PFA for 1 h. After being washed three times with PBS, the cells were incubated with anti-FLAG (1:1000), anti-NS80 (1:500), or anti-NS38 (1:500), followed by incubation with Alexa Fluor 488 conjugated secondary Ab against mouse IgG (1:400) and Alexa Fluor 594 conjugated secondary Ab against rabbit IgG (1:400).

To determine the role of gcTrim47 or its domain mutants on the production of VIBs during GCRV infection, CIK cells (24-well plates, 2.5 × 10^5^ per well) were transfected with 500 ng indicated plasmid for 24 h, and then exposed to GCRV-I (MOI = 1) for 24 h or left untreated. Then, the cells were washed twice with PBS and fixed with 4% PFA for 1 h. After being washed three times with PBS, the cells were incubated with anti-FLAG (1:1000), anti-NS80 Ab (1:500), or antiNS38 Ab (1:500), followed by incubation with Alexa Fluor 488 conjugated secondary Ab against mouse IgG (1:400) and Alexa Fluor 594 conjugated secondary Ab against rabbit IgG (1:400). DAPI staining was applied to detect the cell nucleus. After each incubation step, cells were washed with PBS. Finally, the coverslips were washed, and the images were obtained using an SP8 Leica laser confocal microscopy imaging system.

To further investigate the potential association between gcTrim47 and cellular autophagy activation, CIK cells (24-well plates, 2.5×10^5^ cells per well) were transfected with 500 ng of plasmids encoding gcLC3-GFP, gcTrim47-FLAG, or its deletion mutants (ΔRING, ΔBbox, and ΔSPRY). After 48 hours of transfection, cells were infected with GCRV-I (MOI = 1) for 24 hours or left uninfected. Non-adsorbed virions were removed by PBS washing, and cells were maintained in 2% FBS MEM. Following two additional PBS washes, cells were fixed with 4% PFA for 1 hour. After three PBS rinses, cells were incubated with anti-FLAG primary antibody (1:1000) followed by Alexa Fluor 594-conjugated anti-mouse IgG secondary antibody (1:400). Samples were visualized using confocal microscopy. Autophagosomes were identified as GFP-LC3-labeled puncta, and cells containing ≥5 GFP-LC3-positive puncta were classified as autophagy-activated.

### Co-immunoprecipitation and western blotting analysis

To assess the effect of gcTrim47 and its domain-deletion mutants (gcTrim47-ΔRING, gcTrim47-ΔBbox, gcTrim47-ΔSPRY) on GCRV protein expression, CIK cells (1 × 10^6^ cells per well in 6-well plates) were transfected with plasmids encoding wild-type or mutant gcTrim47 using Lipofectamine 3000 according to the manufacturer’s protocol. Twenty-four hours post-transfection, cells were infected with GCRV at a MOI of 1. Protein lysates were harvested at 24 hpi for downstream analysis.

To assess whether the SPRY domain can independently mediate degradation, CIK cells (1×10^6^ cells/well in 6-well plates) were transfected with increasing concentrations of gcTrim47-SPRY-FLAG plasmids. Twenty-four hours post-transfection, cells were infected with GCRV-I at an MOI of 1, and protein lysates were harvested at 24 hpi for downstream analysis.

To evaluate the impact of gcTrim47 and its domain mutants on autophagy-related p62 protein dynamics, CIK cells (1×10^6^ cells/well) were transfected with 2000 ng of YFP-FLAG (control), gcTrim47-FLAG, gcTrim47-ΔRING-FLAG, gcTrim47-ΔBbox-FLAG, or gcTrim47-ΔSPRY-FLAG plasmids. Following 24 h of transfection, cells were infected with GCRV-I (MOI = 1), and protein samples were collected at 24 hpi for immunoblot analysis of p62 expression.

To dissect the molecular mechanism underlying gcTrim47-mediated GCRV protein degradation, transfected CIK cells were pre-treated with 40 μM MG132 (proteasome inhibitor), 40 mM NH_4_Cl (lysosomal inhibitor), or 10 mM 3-MA (autophagy inhibitor) for 6 hours prior to protein extraction. The amount of additive was determined according to previous report (32). These inhibitors were dissolved in sterile PBS and added to culture media at the indicated concentrations.

For Co-IP analysis of interactions between gcTrim47 (wild-type/mutants) and GCRV nonstructural proteins NS38/NS80, CIK cells seeded in 10-cm dishes were transfected with plasmids expressing YFP-FLAG (negative control), gcTrim47-FLAG, or its deletion mutants. At 24 hours post-transfection, cells were infected with GCRV (MOI = 1), followed by treatment with 20 mM 3-MA at 18 hpi to inhibit autophagic flux or left untreated. Cellular lysates were prepared at 24 hpi using 600 μL ice-cold IP buffer containing protease inhibitor cocktail. Debris was removed by centrifugation at 12,000 × g for 10 min at 4°C. Co-IP was performed using an anti-FLAG M2 magnetic bead kit: clarified lysates were incubated with pre-washed beads at 4°C overnight with gentle rotation. Beads were washed six times with ice-cold wash buffer, and bound proteins were eluted with 1× sodium dodecyl sulfate (SDS) loading buffer at 95°C for 10 min.

Western blotting was conducted as follows: whole-cell extracts or IP eluates were separated by 10% sodium dodecyl sulfate-polyacrylamide gel electrophoresis (SDS-PAGE) and electrotransferred to polyvinylidene fluoride (PVDF) membranes. Membranes were blocked with 5% non-fat dry milk in TBST (20 mM Tris-HCl, pH 7.5, 150 mM NaCl, 0.1% Tween-20) for 1 hour at room temperature, then incubated with primary antibodies—anti-GAPDH (1:5,000), anti-FLAG (1:5,000), anti-NS80 (1:5,000), anti-NS38 (1:5,000), anti-VP3 (1:5,000), anti-VP5 (1:5,000)—overnight at 4°C with gentle agitation. After three TBST washes, membranes were probed with HRP-conjugated goat anti-mouse or anti-rabbit secondary antibodies (1:5,000) for 1 hour at room temperature. Protein bands were visualized using Pierce ECL Western Blotting Substrate and detected with a LAS 4000mini chemiluminescence imaging system.

### Immunization and infection of grass carp

To evaluate the protective efficacy of gcTrim47-expressing engineered *S. cerevisiae* against GCRV-II infection in grass carp, the following experimental protocol was adopted. Fish were immunized via two intraperitoneal injections (200 μL each) of either the gcTrim47-expressing engineered yeast strain or the control PYD1 strain (1.2×10^8^ colony-forming units [CFU]/mL), with a two-week interval between administrations. Thirty days post-immunization, 105 fish per group were infected with GCRV-II (1.38×10^9^ copies/μL) and maintained in 70-L tanks containing 30 L of well-aerated water at 28 ± 1°C. Survival rates were monitored daily for 14 dpi, and survival curves were generated using GraphPad Prism 6 software with statistical significance assessed by log-rank test.

At 48 and 72 hpi, three fish per group were euthanized, and tissue samples (liver, spleen, and kidney) were collected for downstream analyses. These samples including liver, spleen and kidney were used to quantify viral loads via quantitative reverse transcriptase PCR (qRT-PCR), while spleen and kidney tissues were processed for hematoxylin and eosin (H&E) staining.

For constitutive expression analysis of gcTrim47, tissues from 9 healthy fish—including brain, heart, intestine, muscle, spleen, liver, gill, skin, blood, and kidney—were collected and subjected to qRT-PCR. For inducible expression analysis, liver, spleen, and kidney tissues were harvested at 6, 12 and 24 hpi from two groups: 9 grass carp injected with PBS (control) and 9 grass carp infected with GCRV-II (1.38×10^9^ copies/μL), followed by qRT-PCR analysis.

### RNA extraction, reverse transcription, and qRT-PCR

Total RNA was extracted from the liver, kidney and spleen of grass carp collected at 48 and 72 hpi with GCRV-II infection using the Trizol reagent (Invitrogen). The cDNA was synthesized using the RevertAid First Stand cDNA Synthesis Kit (Thermo Fisher Scientific). qRT-PCR was performed using iQ SYBR Green Supermix (Bio-Rad, Singapore) on a Bio-Rad CFX96 Real-Time system under the following conditions: 95°C for 3 min, followed by 45 cycles of 95°C for 10 s, 56–60°C for 20 s, and 72°C for 30 s. The housekeeping genes, including GAPDH and EF-1α, were used to normalize cDNA amounts. The fold changes were calculated based on the 2^−ΔΔCt^ method. All primers used for qRT-PCR are listed in **Table 1**.

### Histopathological observation

The tissue samples preserved in 10% formalin were cutted into blocks, dehydrated through a graded ethanol series, and cleared in xylene. The tissues were then embedded in molten paraffin. After the paraffin blocks solidified, they were trimmed and sectioned. The resulting paraffin sections were mounted onto clean glass slides, dried, and then subjected to deparaffinization, rehydration, and hematoxylin and eosin (H&E) staining. Finally, the stained sections were coverslipped with neutral balsam, dried, and examined under a light microscope to observe the pathological changes, with photomicrographs taken for documentation.

### Statistical analysis

Statistical analysis and graphs were performed and produced using GraphPad Prism 7.0 software. Data from qRT-PCR are presented as mean and SEM. The significance of results was analyzed by an ANOVA or Student’s t-test (*p < 0.05, **p < 0.01, ns, not significant).

## Results

### Overexpression of gcTrim47 inhibits GCRV-I infection and replication *in vitro*

**Figure 1:**
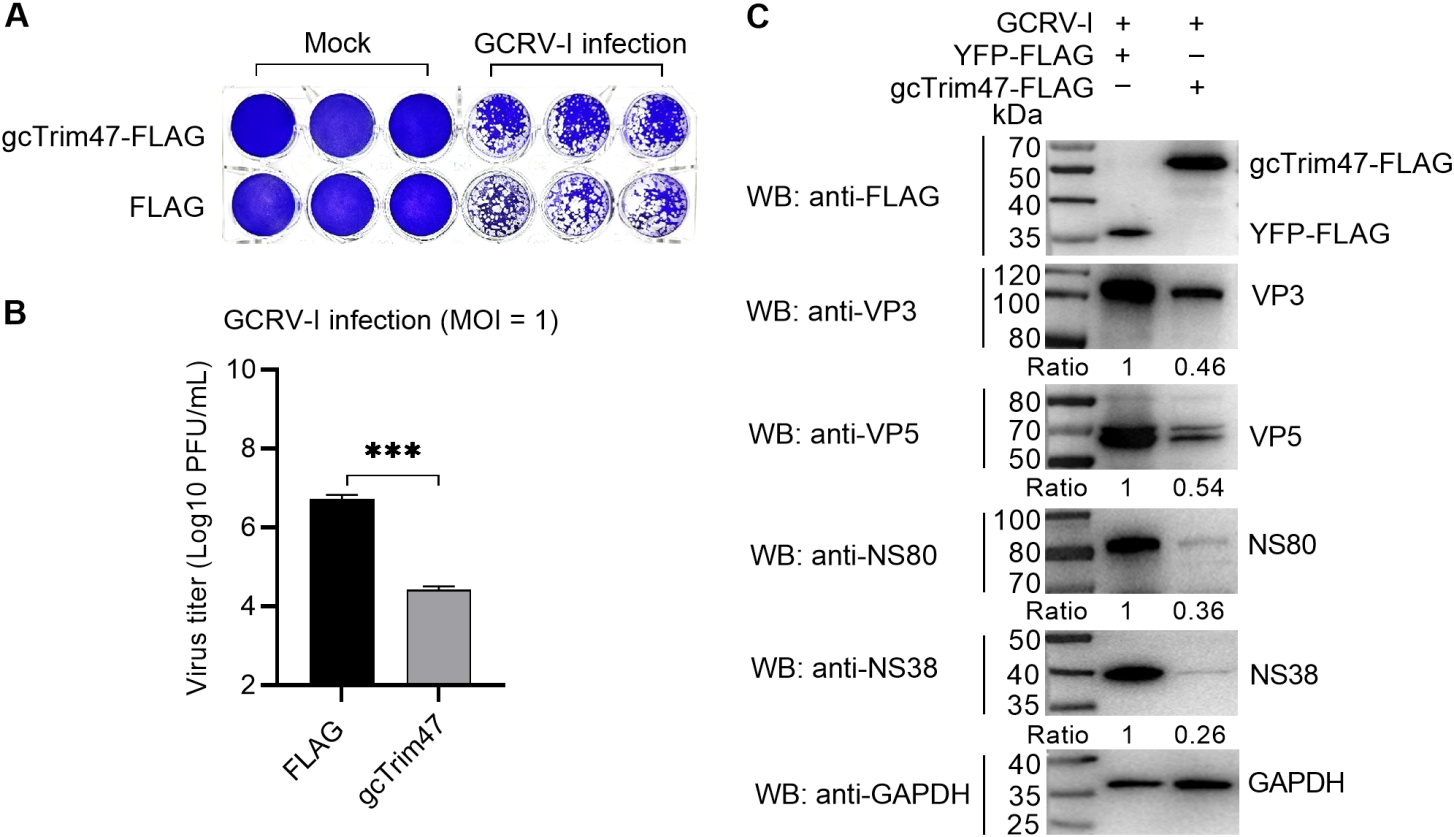
Effect of gcTrim47 on GCRV replication. **(A, B)** Crystal violet staining **(A)** and plaque assay-based quantification of GCRV titers **(B)** were performed in CIK cells transfected with FLAG or gcTrim47-FLAG and infected with GCRV at a MOI of 1. **(C)** Expression levels of GCRV structural proteins (VP3, VP5) and nonstructural proteins (NS38, NS80) were analyzed in CIK cells transfected with FLAG or gcTrim47-FLAG and infected with GCRV at a MOI of 1. Protein band intensities were quantified using Image J software for densitometric analysis. ***p < 0.001.

To characterize the *in vitro* role of gcTrim47 in GCRV infection, CIK cells were transfected with plasmids encoding gcTrim47-FLAG or empty FLAG vector, followed by infection with GCRV-I at a MOI of 1. Crystal violet staining and plaque assay analyses revealed that, compared with the empty vector control, gcTrim47-overexpressing cells maintained significantly higher viability and exhibited a reduction in viral titers at 24 hpi, indicating that gcTrim47 potently restricts GCRV-I replication in CIK cells (**Figures 1A, B**).

To further investigate the impact of gcTrim47 on GCRV protein expression, Western blot analysis was performed using cell lysates from gcTrim47-FLAG or YFP-FLAG transfected cells at 24 hpi. Detection of viral structural proteins (VP3, VP5) and nonstructural proteins (NS38, NS80) revealed that gcTrim47 overexpression significantly attenuated the accumulation of all tested viral proteins compared to the control group (**Figure 1C**).

These findings collectively demonstrate that gcTrim47 exerts antiviral activity by disrupting both structural and nonstructural protein synthesis, thereby impairing GCRV-I propagation in fish cells.

### gcTrim47 degrades the nonstructural proteins NS38 and NS80 in a SPRY domain-dependent manner

**Figure 2:**
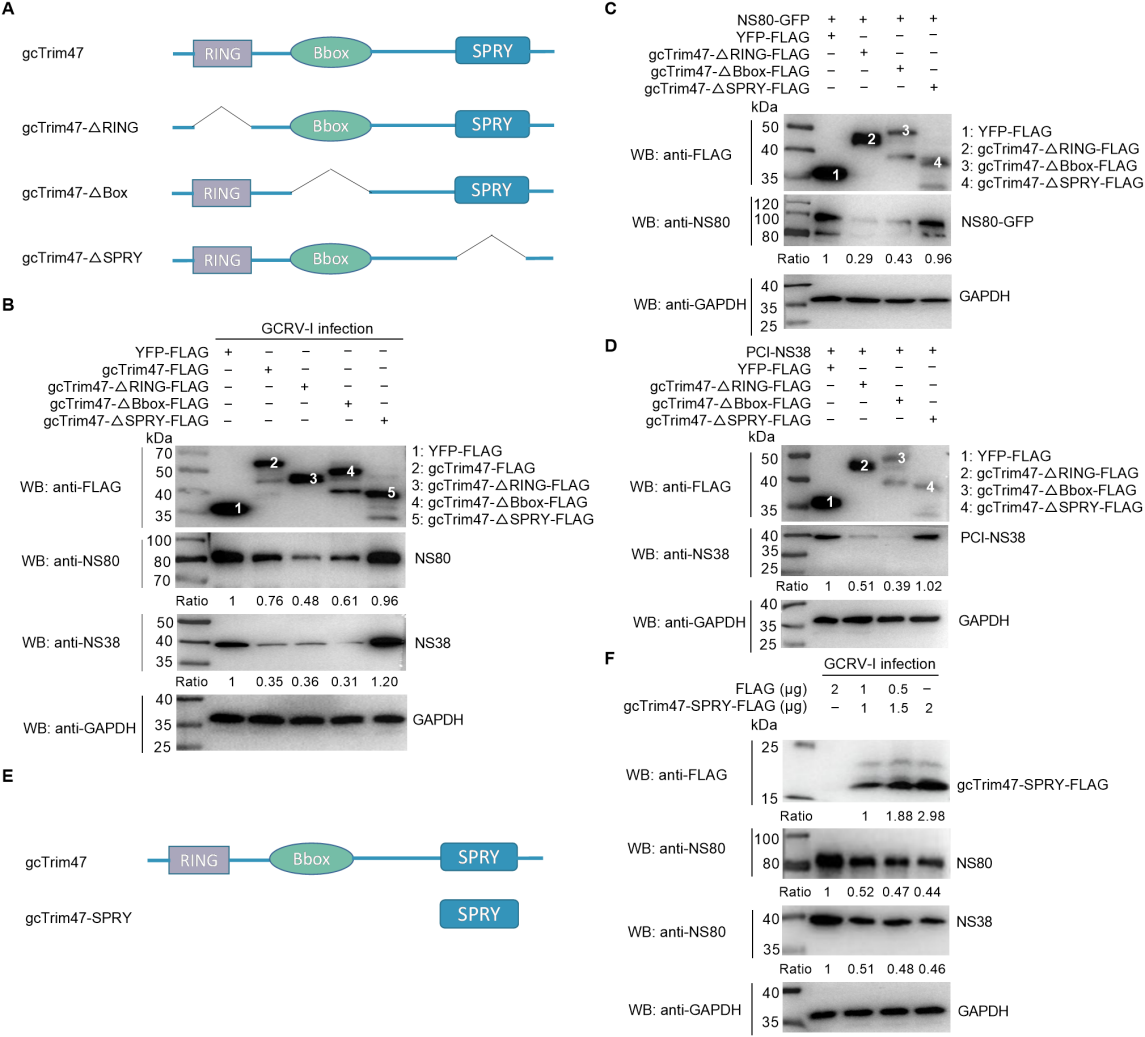
gcTrim47 degrades GCRV non-structural proteins in a SPRY domain-dependent manner. **(A)** Schematic diagrams of different domain-deletion constructs of gcTrim47. **(B)** The effect of different domain-deletion constructs of gcTrim47 on the endogenous expression of GCRV non-structural proteins NS80 and NS38. **(C, D)** The effect of different domain-deletion constructs of gcTrim47 on the exogenous expression of GCRV non-structural proteins NS80 or NS38. **(E)** Schematic diagrams of gcTrim47 and gcTrim47-SPRY (a mutant containing only the SPRY domain). **(F)** The effect of gcTrim47-SPRY on the endogenous expression of GCRV non-structural proteins NS80 and NS38. Protein band intensities were quantified using Image J software for densitometric analysis.

The gcTrim47 protein comprises an N-terminal RING zinc-finger domain, a B-box domain, and a C-terminal PRY/SPRY domain, with the characteristic features of the TRIM family’s C-VII subgroup. The highly divergent PRY/SPRY domain typically dictates substrate specificity among TRIM family members, prompting us to investigate its role in GCRV interactions. We generated three domain-deletion constructs: gcTrim47-ΔRING-FLAG (RING domain deleted), gcTrim47-ΔBbox-FLAG (B-box domain deleted), and gcTrim47-ΔSPRY-FLAG (SPRY domain deleted), confirmed by Sanger sequencing (**Figure 2A**). These plasmids, along with full-length gcTrim47-FLAG, were transiently overexpressed in CIK cells. Immunoblot analysis revealed that overexpression of wild-type gcTrim47-FLAG, gcTrim47-ΔRING-FLAG, and gcTrim47-ΔBbox-FLAG significantly reduced endogenous levels of GCRV-I nonstructural proteins NS80 and NS38 (**Figure 2B**).

Co-transfection with gcTrim47 domain-deletion constructs and NS80-GFP or PCI-NS38 enabled assessment of protein-protein interactions and viral protein regulation. Immunoblot analysis revealed that overexpression of gcTrim47-ΔRING-FLAG and gcTrim47-ΔBbox-FLAG significantly reduced exogenous levels of GCRV-I nonstructural proteins NS80 and NS38 (**Figures 2C, D**). Strikingly, however, the gcTrim47-ΔSPRY mutant failed to suppress either endogenous or exogenous viral protein levels (**Figures 2B–D**), establishing the SPRY domain as essential for gcTrim47-mediated degradation of GCRV-I NS38 and NS80.

To assess whether the SPRY domain alone is sufficient to recapitulate the degradation activity of full-length gcTrim47, a mutant containing only the SPRY domain (gcTrim47-SPRY-FLAG) was constructed (**Figure 2E**). Immunoblot analysis demonstrated that overexpression of gcTrim47-SPRY-FLAG at varying concentrations significantly reduced endogenous NS38 and NS80 levels in a manner dependent on protein expression dose. While the endogenous levels of NS38 and NS80 gradually decreased with increasing gcTrim47-SPRY-FLAG abundance, the dose-dependent effect exhibited a non-linear relationship (**Figure 2F**). These results indicate that the SPRY domain alone can mediate viral protein degradation.

### gcTrim47 degrades the nonstructural proteins NS38 and NS80 via the autophagy pathway

**Figure 3:**
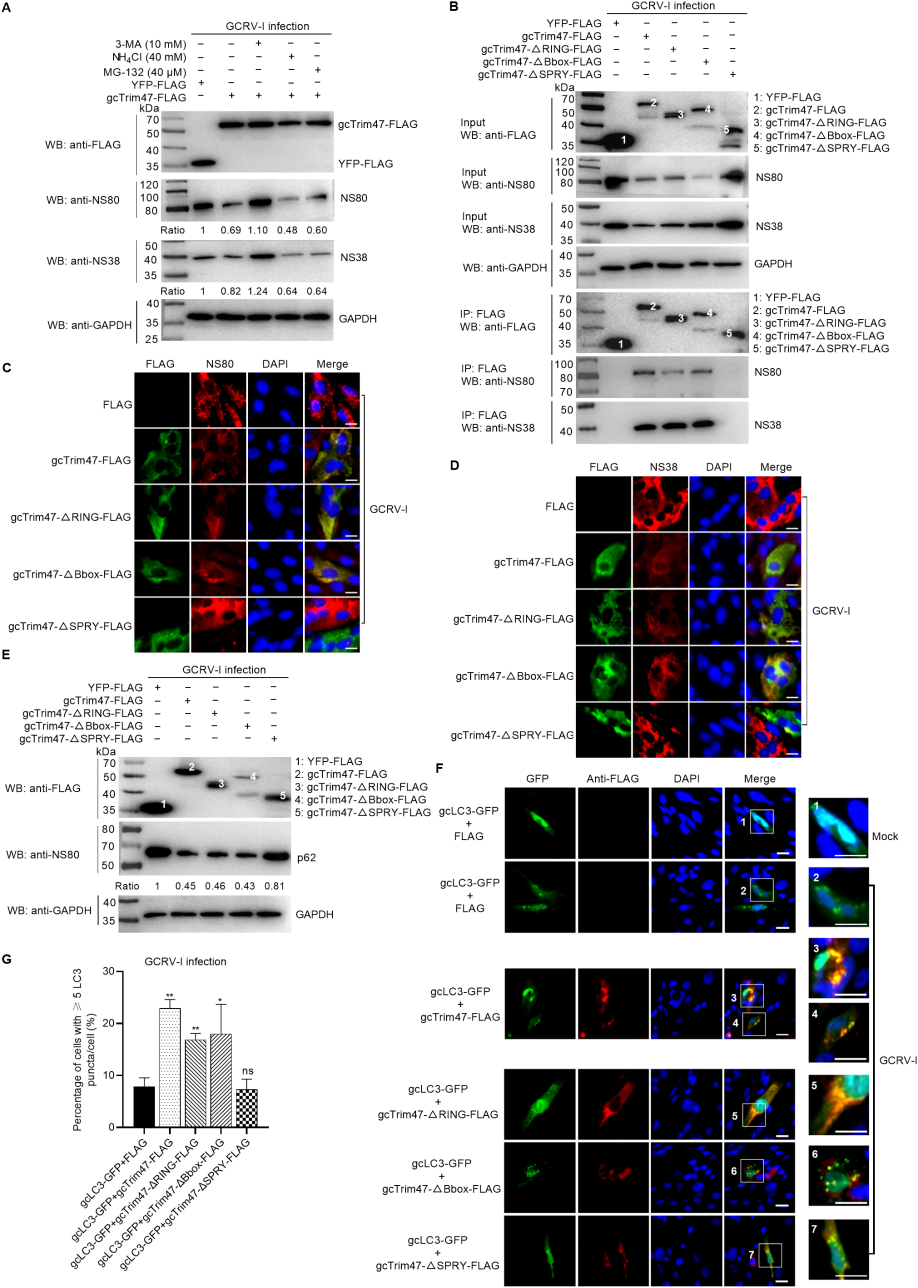
gcTrim47 promotes degradation of GCRV nonstructural proteins through the autophagy pathway. **(A)** Evaluation of gcTrim47-mediated degradation of GCRV nonstructural proteins NS80 and NS38 in the presence of ubiquitin-proteasome (MG-132) and autophagy-lysosome (3-MA and NH_4_Cl) pathway inhibitors. **(B)** Interaction analyses between full-length gcTrim47 or its domain-deleted constructs and GCRV nonstructural proteins in CIK cells infected with GCRV-I. **(C, D)** Confocal microscopy analysis of subcellular co-localization between gcTrim47/domain-deleted constructs and GCRV nonstructural proteins NS80 **(C)** or NS38 **(D)** in GCRV-infected CIK cells. **(E)** The effect of different domain-deletion constructs of gcTrim47 on the endogenous expression of p62. **(F)** The subcellular colocalization between gcTrim47/domain-deleted constructs and autophagosomes (labeled by gcLC3-GFP). **(G)** The effect of different domain-deletion constructs of gcTrim47 on the autophagosome formation. Protein band intensities were quantified using Image J software for densitometric analysis.

To dissect the molecular pathway by which gcTrim47 facilitates degradation of GCRV-I nonstructural proteins NS38 and NS80, we employed pharmacological inhibitors targeting major proteolytic systems in CIK cells overexpressing gcTrim47-FLAG: MG132 (ubiquitin-proteasome pathway inhibitor), 3-MA (autophagy pathway inhibitor), and NH_4_Cl (lysosomal pathway inhibitor). Immunoblot analysis revealed that treatment with MG132 or NH_4_Cl had no impact on gcTrim47-mediated reduction of NS80/NS38 levels. However, 3-MA completely restored viral protein expression to control levels (**Figure 3A**). These results indicate that gcTrim47-dependent degradation of NS80/NS38 is strictly dependent on the autophagy pathway.

To determine whether gcTrim47 and its domain mutants directly engage NS80/NS38 for degradation, Co-IP assays were performed in CIK cells transfected with gcTrim47-FLAG or its deletion variants under conditions of 3-MA treatment to block autophagic flux. Endogenous NS38 and NS80 were pulled down with gcTrim47-FLAG, gcTrim47-ΔRING-FLAG, gcTrim47-ΔBbox-FLAG, and gcTrim47-ΔSPRY-FLAG (**Supplementary Figure S1A**). Notably, in the absence of 3-MA—where autophagic degradation proceeds unimpeded—gcTrim47-ΔSPRY-FLAG failed to exhibit detectable interaction with NS38/NS80 (**Figure 3B**), highlighting the SPRY domain’s indispensable role in substrate recognition and gcTrim47-NS38/NS80 interaction.

Subcellular colocalization studies further illuminated the spatiotemporal dynamics of this interaction. Under 3-MA treatment, gcTrim47-FLAG and all mutants displayed significant cytoplasmic punctate colocalization with NS80/NS38 during GCRV-I infection (**Supplementary Figures S1B, C**). Conversely, in the absence of 3-MA, only wild-type gcTrim47 and its ΔRING/ΔBbox mutants retained colocalization with viral proteins, while the ΔSPRY mutant showed no appreciable overlap (**Figures 3C, D**). These results underscore the critical role of the SPRY domain in targeting gcTrim47 to NS38/NS80-containing compartments.

To investigate the role of gcTrim47 and its domain mutants in autophagy regulation, Western blotting was employed to assess their impact on p62 (SQSTM1) protein expression, while immunofluorescence microscopy was used to evaluate their subcellular colocalization with autophagosomes (labeled by LC3) and effects on autophagosome formation. Under GCRV-I infection, gcTrim47 wild-type, ΔRING, and ΔBbox mutants (all retaining an intact SPRY domain) significantly reduced p62 levels, indicative of enhanced autophagic flux, whereas the ΔSPRY mutant—lacking the SPRY domain—exhibited no significant effect on p62 degradation (**Figure 3E**). Immunofluorescence analysis revealed that all gcTrim47 variants, including ΔSPRY, displayed significant colocalization with gcLC3-positive autophagosomes during GCRV-I infection (**Figure 3F**), suggesting that the SPRY domain is not required for autophagosome targeting. However, quantitative analysis of autophagosome activation showed that only gcTrim47 wild-type, ΔRING, and ΔBbox mutants—with intact SPRY domains—significantly increased the proportion of gcLC3-positive cells in GCRV-I infected cells. In contrast, the ΔSPRY mutant failed to induce the increase of the activated autophagosome despite retaining the RING and Bbox domains (**Figure 3G**), highlighting the SPRY domain’s critical role in linking substrate recognition to autophagic flux activation.

These findings establish a mechanistic hierarchy: the SPRY domain mediates specific engagement with viral proteins (NS38/NS80), enabling gcTrim47 to promote autophagosome formation and p62-dependent cargo clearance, while the RING/Bbox domains may modulate autophagic efficiency but are insufficient to drive substrate-specific degradation in the absence of SPRY.

### gcTrim47 impairs the production of VIBs and GCRV-I replication in a SPRY domain-dependent manner

The above-mentioned experiments demonstrated that gcTrim47 restricted GCRV-I infection and replication while reducing expression of the nonstructural proteins NS38 and NS80 (**Figure 1**). Given that these proteins are essential for forming VIBs, we hypothesized that gcTrim47 might disrupt VIBs formation. To test this, we transiently overexpressed wild-type gcTrim47-FLAG, gcTrim47-ΔRING-FLAG, gcTrim47-ΔBbox-FLAG, or gcTrim47-ΔSPRY-FLAG mutants in 24-well plates and visualized VIBs formation via immunofluorescence staining for NS38/NS80.

**Figure 4:**
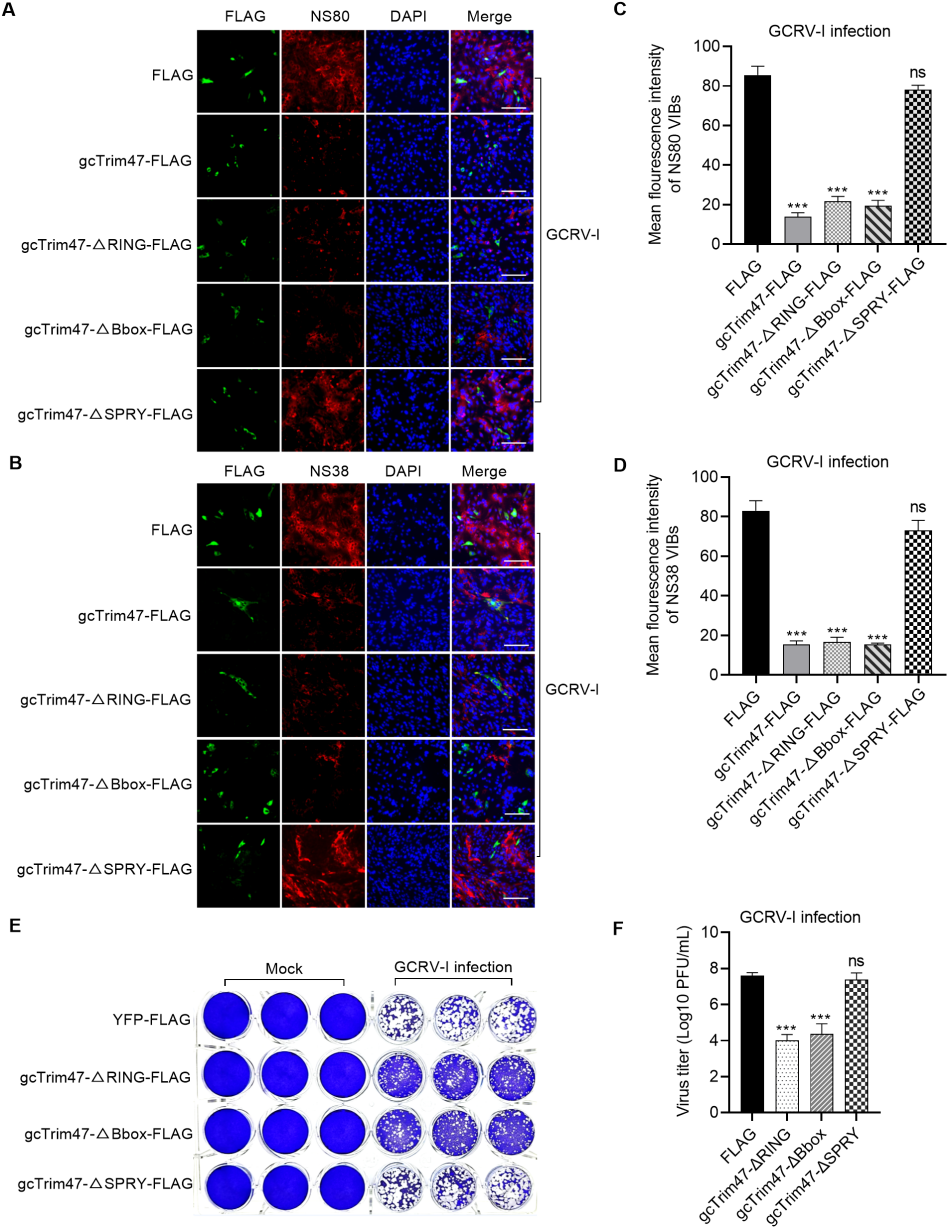
gcTrim47 inhibits the formation of VIBs and GCRV infection in a SPRY domain-dependent manner. **(A, B)** Immunofluorescence analysis of VIB formation using anti-NS80 **(A)** or anti-NS38 **(B)** antibodies, evaluating the impact of full-length gcTrim47 and its domain-specific mutants. **(C, D)** Quantitative analysis of average VIB fluorescence intensity, assessed via anti-NS80 **(C)** or anti-NS38 **(D)** antibody staining. **(E, F)** The effect of different domain-deletion constructs of gcTrim47 on the GCRV infection, assessed via crystal violet staining **(E)** and plaque assay-based quantification of GCRV titers **(F)**. ***p < 0.001.

Compared with the empty vector control, cells expressing wild-type gcTrim47, gcTrim47-ΔRING, or gcTrim47-ΔBbox displayed significant reductions in both the numbers and sizes of NS38/NS80-positive VIBs. In contrast, gcTrim47-ΔSPRY overexpression had no discernible effect on VIBs formation (**Figures 4A-D**). These results corroborate the critical role of the SPRY domain: even without the RING or B-box domain, gcTrim47 retains the ability to disrupt VIBs production, whereas SPRY deletion abolishes this function.

We next investigated whether gcTrim47 restricted GCRV-I infection and replication via the SPRY domain. CIK cells were transfected with gcTrim47-ΔRING-FLAG, gcTrim47-ΔBbox-FLAG, or gcTrim47-ΔSPRY-FLAG mutants in 24-well plates. The results from crystal violet staining showed that gcTrim47 lacking the RING or B-box domain retained antiviral function, whereas SPRY deletion abrogated its anti-GCRV-I activity (**Figure 4E**). gcTrim47 also influenced GCRV-I replication through its SPRY domain rather than its RING or B-box domain (**Figure 4F**).

Collectively, these findings demonstrate that gcTrim47 mediates antiviral activity specifically via its SPRY domain, targeting the formation of VIBs to disrupt the viral replication machinery and inhibit GCRV infection.

### Establishment of the gcTrim47-PYD1/EBY100 yeast surface display system and grass carp GCRV-II infection

**Figure 5:**
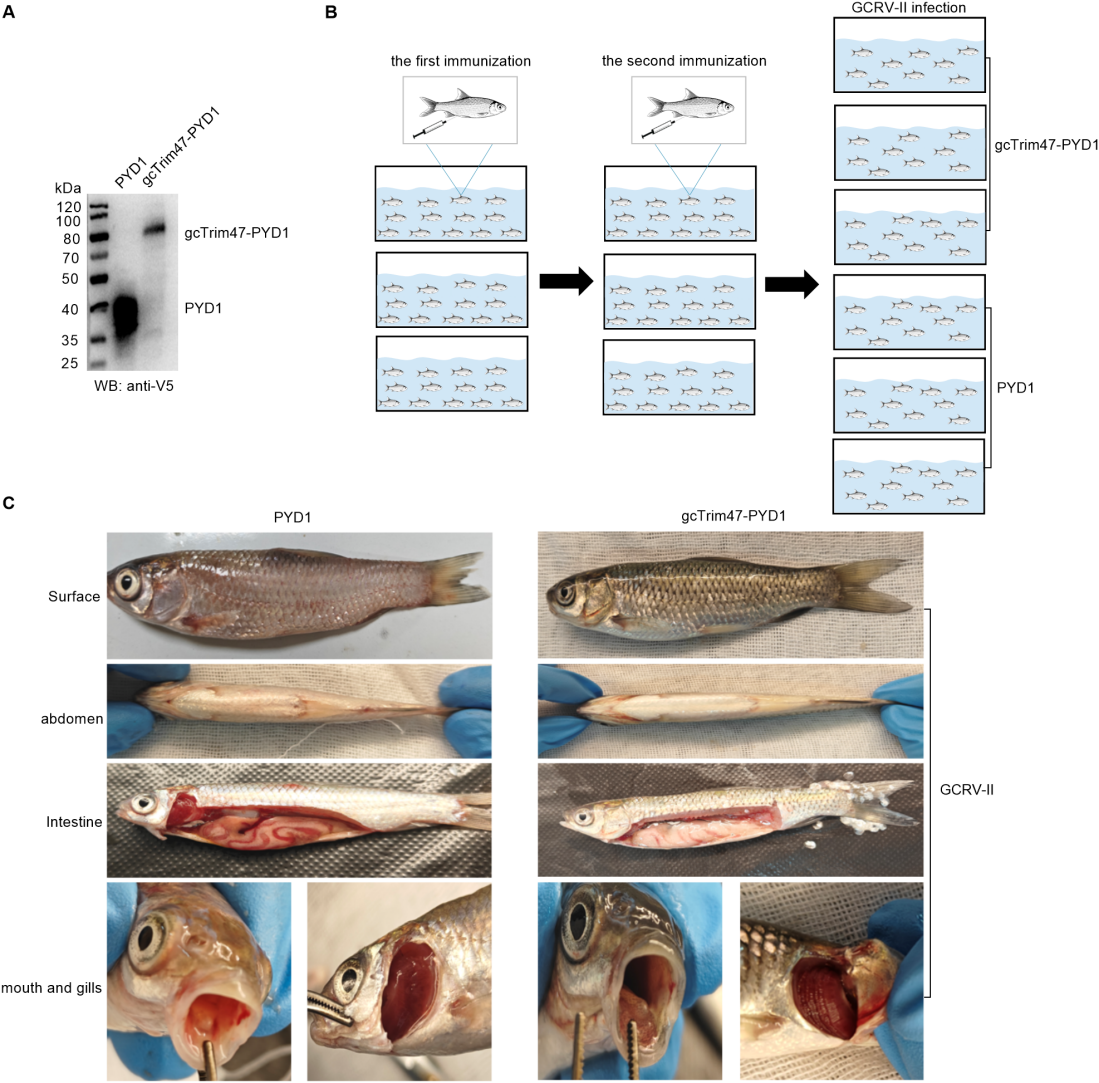
Construction of the gcTrim47-PYD1/EBY100 yeast surface display system and *in vivo* GCRV-II infection model. **(A)** Western blot analysis of cell lysates from *S. cerevisiae* EBY100 expressing either PYD1 or gcTrim47-PYD1, confirming fusion protein expression. **(B)** Schematic depiction of the intraperitoneal immunization protocol followed by GCRV-II challenge in grass carp. **(C)** Comparative analysis of clinical manifestations in grass carp following GCRV-II infection.

The gcTrim47-PYD1 fusion construct was inserted downstream of the GAL1 and T7 promoters in the *Saccharomyces cerevisiae* expression vector. D-galactose was used to induce the expression of gcTrim47-PYD1. Cell lysates were analyzed by 10% SDS- PAGE. Western blotting, with an anti-V5 antibody probing the lysates from cells transfected with the gcTrim47-PYD1 plasmid, detected a distinct protein band around 80 kDa (**Figure 5A**). This Western blot result demonstrates the successful expression of gcTrim47-PYD1 in the *Saccharomyces cerevisiae* strain.

To explore the potential immune-protective effects of gcTrim47-expressing engineered *Saccharomyces cerevisiae* in grass carp, grass carp were immunized with the prepared yeast - based immunogen. After two rounds of immunization, the fish were challenged with GCRV-II by injection (**Figure 5B**). At the time of infection, 105 fish were randomly selected and divided into two groups, and each group was further divided into three replicates for the GCRV-II infection.

One week after intraperitoneal injection of GCRV-II, grass carp began to show disease symptoms, indicating successful infection with GCRV-II. The symptomatic grass carp were dissected for further analysis. A comparison of the clinical symptoms between the two groups is as follows. As shown in the images, compared to the experimental group, the control group had more severe hemorrhage and redness on the body surface, abdomen, intestines, oral cavity, and gills. Although the experimental group also exhibited typical symptoms of hemorrhagic disease, the severity was significantly milder (**Figure 5C**).

### Protective effects of the engineered *S. cerevisiae* expressing gcTrim47 in the grass carp against GCRV-II infection

**Figure 6:**
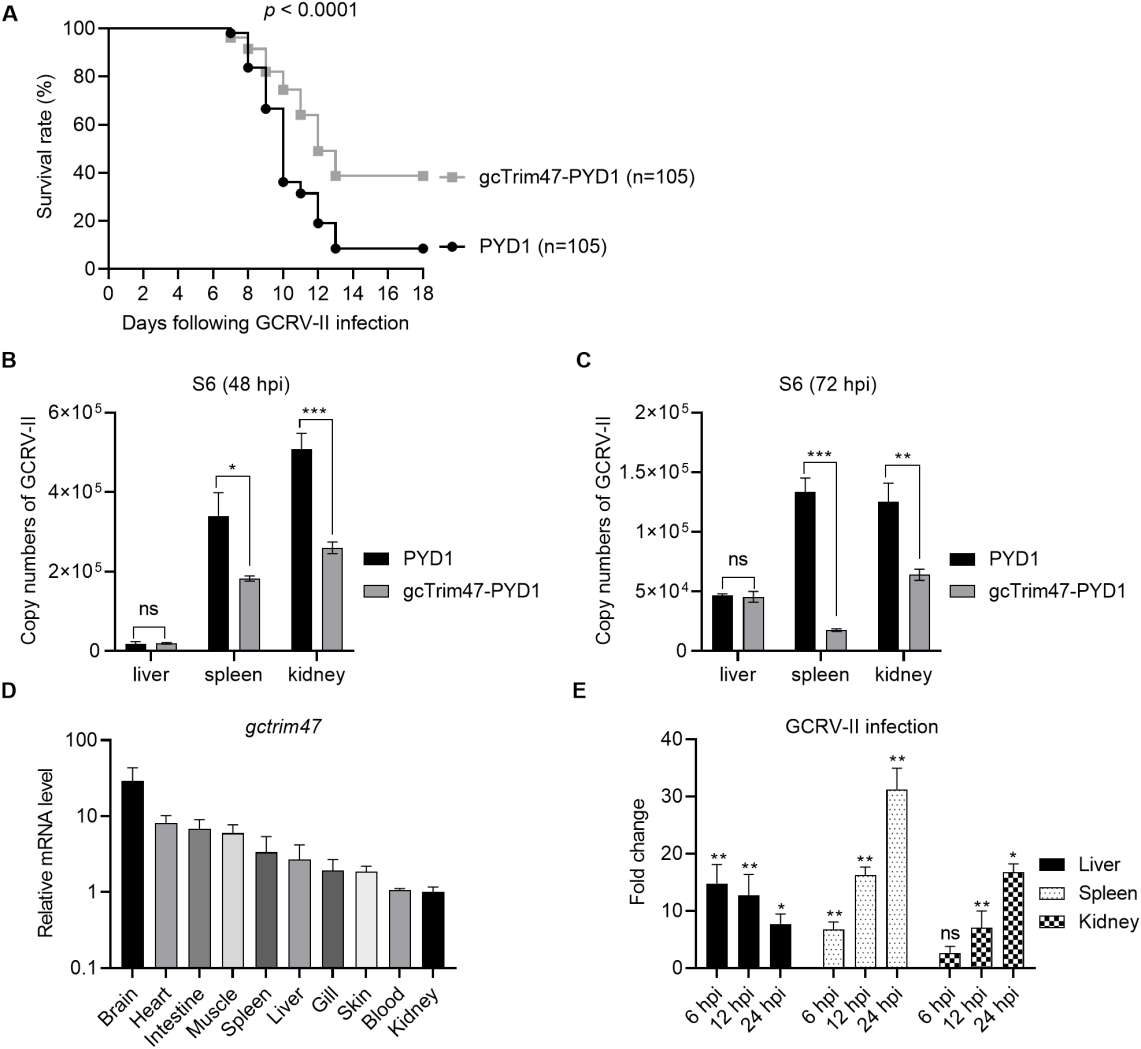
Protective efficacy of engineered *S. cerevisiae* expressing gcTrim47 against GCRV-II infection in grass carp. **(A)** Assessment of the survival rate of grass carp following GCRV-II infection, comparing groups treated with engineered *S. cerevisiae* expressing gcTrim47 and control groups. **(B, C)** Quantification of the GCRV-II S11 gene copy numbers in the liver, spleen, and kidney tissues of grass carp. These tissues were collected from fish immunized with PYD1 or gcTrim47-PYD1 and infected with GCRV-II. **(D)** The constitutive expression of gcTrim47 in different tissues, including brain, heart, intestine, muscle, spleen, liver, gill, skin, blood and kidney. **(E)** The inducible expression of gcTrim47 in the liver, spleen, and kidney from grass carp infected with GCRV-II. These 3 tested tissues were collected at 6, 12 and 24 hpi. *p < 0.05, **p < 0.01, ***p < 0.001.

Grass carp immunized with gcTrim47-expressing engineered *S. cerevisiae* (gcTrim47-PYD1/EBY100) and control yeast (PYD1/EBY100) were infected with GCRV-II via immersion exposure (1.38×10^9^ copies/mL). Mortality was monitored daily for 18 dpi, with cumulative mortality recorded. The control group exhibited a high cumulative mortality of 91.43%, whereas the gcTrim47-PYD1/EBY100 group showed a significantly reduced mortality rate of 61.32%, yielding a calculated protective efficiency of 32.94% using the standard formula for relative percent survival (RPS) (**Figure 6A**). These data confirm that gcTrim47-expressing yeast confers substantial protection against GCRV-II infection in grass carp.

Viral load analysis was performed at 48 and 72 hpi. Liver, spleen, and kidney tissues were collected from both groups for qRT-PCR to assess GCRV-II copies using S6 primer. Control fish (PYD1/EBY100) displayed significantly higher viral titers in spleen and kidney tissues compared to the gcTrim47-PYD1/EBY100 group, indicating effective restriction of viral dissemination. Conversely, no significant difference in hepatic viral load was observed between groups, suggesting tissue-specific antiviral activity (**Figures 6B, C**).

To characterize whether the tissue-specific expression pattern of gcTrim47 exist, its distribution across multiple tissues, including brain, heart, intestine, muscle, spleen, liver, gill, skin, blood and kidney, was analyzed by qRT-PCR. Results revealed constitutive expression of gcTrim47 in all tested tissues, with the highest transcript levels observed in brain tissue, followed by heart and intestine. Notably, kidney tissue exhibited the lowest basal expression among those assayed (**Figure 6D**). Further investigation focused on the inducible expression of gcTrim47 in immune-relevant organs (liver, spleen, and kidney). Following GCRV-II infection, significant upregulation of gcTrim47 mRNA was detected in all three tissues, indicating a dynamic response to viral challenge. Unlike the inducible expression pattern of gcTrim47 in the liver, where the induction fold significantly decreases as infection time prolongs, the expression of gcTrim47 in the spleen and kidney increases continuously (**Figure 6E**).

### Histopathological analysis of the spleen and kidney tissues in grass carp infected with GCRV-II

**Figure 7:**
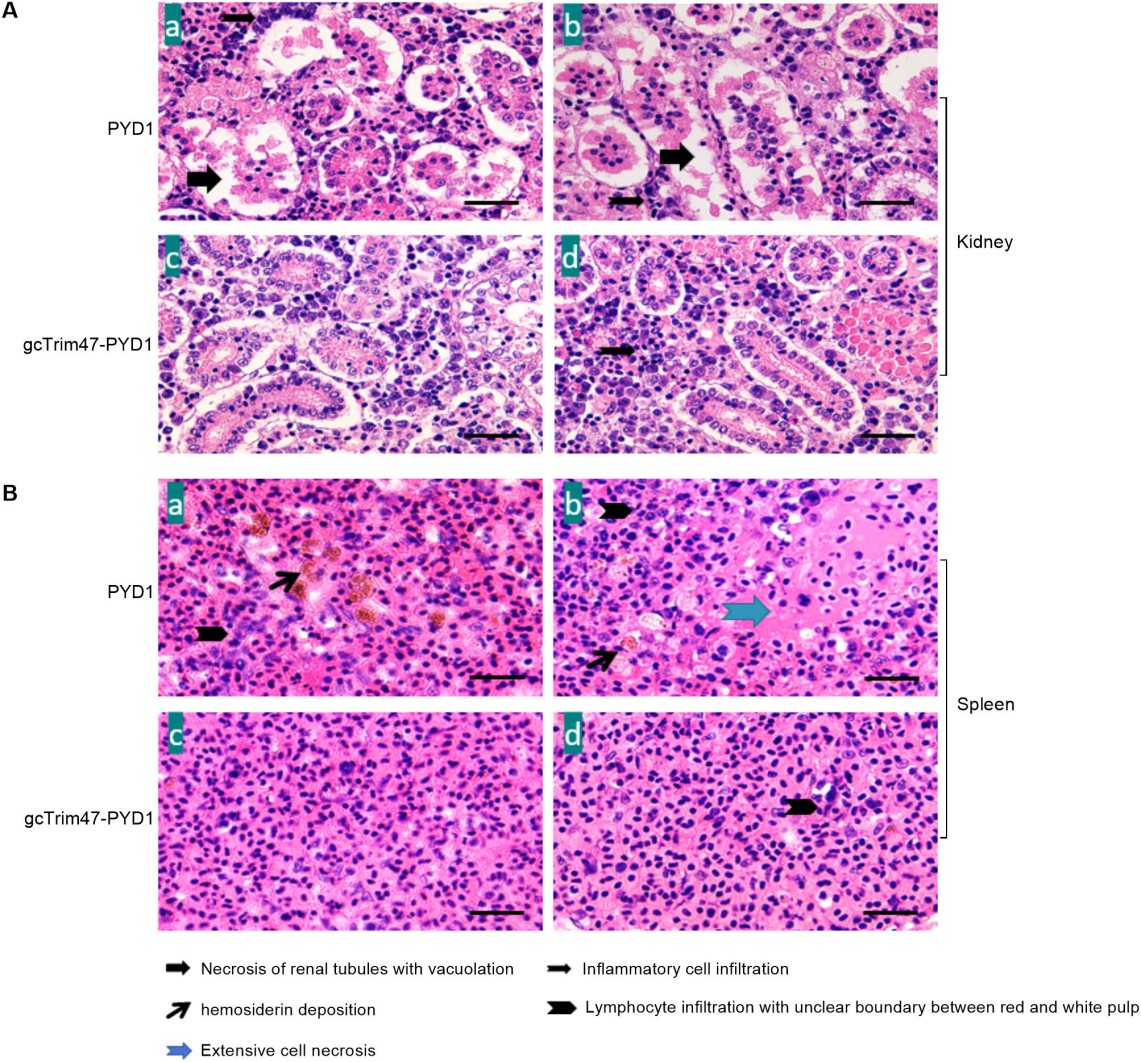
Histopathological evaluation of spleen and kidney tissues in GCRV-II-infected grass carp. **(A)** Renal histology of GCRV-II-infected grass carp; **(B)** Splenic histology of GCRV-II-infected grass carp. (a, b) PYD1 control group; (c, d) gcTrim47-PYD1 immune group.

Histopathological examination of renal tissue revealed marked structural differences between groups. In the PYD1 control group, kidney sections exhibited disorganized architecture with extensive parenchymal necrosis, severe tubular vacuolization, and prominent interstitial hemorrhage accompanied by inflammatory cell infiltration (**Figures 7A**-a, **A**-b). In contrast, the gcTrim47-PYD1 group showed preserved renal tubule morphology, minimal inflammatory cell infiltration, and organized tissue architecture with no evidence of vacuolization or necrosis (**Figures 7A**-c, **A**-d).

Spleen tissue analysis further highlighted the protective effect of gcTrim47-expressing yeast. Control group sections displayed large foci of cellular necrosis, interfollicular hemorrhage, and prominent hemosiderin deposits within the red pulp (**Figures 7B**-a, **B**-b). Conversely, the gcTrim47-PYD1 group exhibited well-preserved splenic architecture with distinct demarcation between red and white pulp, orderly cell arrangement, and absence of significant hemosiderin deposition or necrotic foci (**Figures 7B**-c, **B**-d).

## Discussion

The innate immune system serves as the initial line of defense for the host against pathogen invasion. IFN production triggered by the RLR signaling pathway plays a crucial role in inhibiting viral infection (33). Over the past decade, accumulating evidences have demonstrated that TRIM proteins modulate the host’s antiviral immune responses via ubiquitination-dependent or autophagy-mediated pathways. This study elucidated the molecular mechanisms by which grass carp TRIM protein combat viral infections, focusing on the antiviral function of gcTrim47 against GCRV-I (*in vitro*) and GCRV-II (*in vivo*). Importantly, we engineered and validated a yeast-based immune enhancer, aiming to establish novel strategies for disease control in aquaculture by enhancing host resistance.

The functional versatility of TRIM proteins stems from domain-specific adaptations across species, enabling specialized interactions with viral or cellular targets. In humans, TRIM5α employs its B30.2 (SPRY) domain to directly recognize HIV capsid proteins, triggering proteasomal degradation and viral capsid disassembly (34). TRIM25 utilizes its RING domain to catalyze K63-linked ubiquitination of RIG-I, promoting its oligomerization and interaction with mitochondrial antiviral signaling protein (MAVS) to amplify IFN-β production (35). Teleost fish exhibit analogous yet species-specific mechanisms: zebrafish FTR36 activates the RIG-I/IRF3/IRF7 axis to combat SVCV and giant salamander iridovirus (GSIV) infection through coordinated functions of its RING and B30.2 domains (36), while Trim2b targets SVCV nucleoprotein (N) and glycoprotein (G) via its NHL_TRIM2_like domain for proteolytic degradation (17). These examples illustrate how domain architecture underlies pathogen-specific recognition across vertebrates.

Mammalian Trim47 has been traditionally linked to oncogenic processes, such as activating NF-κB signaling through its SPRY domain–mediated binding to protein kinase C-ϵ (PKC-ϵ), which drives breast cancer proliferation and endocrine resistance (37). This study, however, uncovers an evolutionary repurposing of the gcTrim47 SPRY domain, which has acquired the ability to selectively recognize GCRV nonstructural proteins NS38 and NS80, targeting them for autophagic elimination. While GCRV hijacks autophagosomes to facilitate VIBs formation and virion assembly as an immune evasion strategy (38), gcTrim47 employs a counteractive “anti-hijacking” mechanism: repurposing the autophagic pathway to degrade viral components essential for VIB biogenesis. Notably, NH_4_Cl (a lysosomal pH neutralizer) fails to block NS38/NS80 degradation, indicating gcTrim47 utilizes autophagosomes as active “sequestration platforms” rather than passive degradation vesicles. Critically, gcTrim47-mediated degradation of these viral proteins occurs independently of lysosomal acidification—a key distinction from canonical autophagy, where lysosomal hydrolases require acidic environments for activity. This proposed mechanism highlights a strategic divergence in autophagic utilization: viruses subvert autophagosomes for replication, while host TRIM proteins exploit autophagic machinery for antiviral defense through non-canonical, lysosome-independent pathways. Previous studies have demonstrated that GCRV VIBs form through liquid-liquid phase separation (LLPS) of the nonstructural protein NS80, which self-assembles into dynamic, membrane-free condensates essential for viral replication (39). We propose two non-canonical degradation mechanisms by which gcTrim47 may counteract GCRV infection: 1) LLPS-driven autophagic sequestration: gcTrim47 leverages its SPRY domain to bind NS80/NS38’s intrinsically disordered regions (IDRs), competing with viral protein self-condensation and redirecting them into host-derived condensates. These condensates act as nucleation sites for autophagosome biogenesis: gcTrim47 recruits the Atg5-Atg12 complex and LC3, promoting phagophore membrane extension to encapsulate viral condensates into “sequestration vesicles”. Degradation within these vesicles may occur via autophagy-related enzymes independent of lysosomal fusion. 2) SPRY-domain-mediated direct destabilization: The SPRY domain of gcTrim47 may directly disrupt NS80/NS38 stability through protein-protein interactions, inducing conformational changes or recruiting cytosolic proteases (e.g., calpains). This mechanism operates independently of canonical lysosomal or proteasomal pathways. These hypothesized pathways—exploiting LLPS for autophagic sequestration or direct SPRY-domain-mediated degradation—warrant further experimental validation to define gcTrim47’s role in non-canonical antiviral autophagy in future.

VIBs, formed post-infection, serve as specialized microcompartments for viral replication and immune evasion. They sequester viral nucleic acids and proteins while shielding them from pattern recognition receptors. For example, Ebola virus VIBs sequester IRF3 to disrupt IFN signaling (40). In GCRV infection, NS80 and NS38 are indispensable for VIB biogenesis, recruiting inner-capsid proteins (VP1, VP4, VP6) and host factors (2, 4). Knockdown of NS38/NS80 impairs viral infectivity (2), while their overexpression triggers VIB formation (31, 41), underscoring their role in pathogenesis. Unlike gcTBK1_tv3, which mediates K48-linked proteasomal degradation of NS80/NS38 (32), gcTrim47 employs autophagy for a dual function: degrading monomeric NS80/NS38 and disrupting VIB assembly. This may arise from TRIM protein oligomerization: the dimeric TRIM5α SPRY domain binds retroviral capsids, with higher-order oligomerization enhancing recognition efficiency (42). Consistently, the gcTrim47 ΔSPRY mutant fails to inhibit replication despite viral protein binding, highlighting the SPRY domain’s critical role in targeting VIB microenvironments.

*S. cerevisiae*, a GRAS (Generally Recognized as Safe)-designated eukaryotic microorganism with a long history of safe use in food fermentation and biopharmaceutical production, serves as an ideal industrial chassis for recombinant protein expression and vaccine development (43, 44). Its regulatory approval by global authorities (e.g., FDA, EFSA) underscores its suitability for producing ingredients in human nutrition and therapeutic applications (45). Mechanistically, *S. cerevisiae* leverages its native secretory pathway—comprising signal peptide-mediated translocation, endoplasmic reticulum (ER) folding, and Golgi glycosylation—to efficiently produce biologically active proteins with correct tertiary structure, a critical advantage over prokaryotic hosts (46, 47). In biopharmaceutical applications, yeast-derived vaccines exhibit superior safety profiles compared to bacterial systems due to the absence of endotoxin contamination and reduced risk of oncogenic transformation relative to mammalian cells. Exploiting the immunomodulatory properties of *S. cerevisiae*, a eukaryotic engineering backbone known to also activate fish innate immunity via cell wall β-glucans and mannanoligosaccharides, we constructed the engineered *S. cerevisiae* expressing gcTrim47-PYD1. In *in vivo* challenges against GCRV-II, this engineered probiotic gcTrim47-PYD1 conferred a RPS of 32.94%, significantly higher than the unvaccinated control group. The cross-species applicability of Trim47 function is particularly intriguing: while its mammalian ortholog functions as a cancer biomarker, the SPRY domain of Trim47 in sensing and degrading viral proteins and immunoprotection capability of gcTrim47-PYD1 in resisting GCRV-II infection demonstrate its possible application as immunoenhancing agents.

In summary, gcTrim47 employs its SPRY domain to selectively degrade GCRV nonstructural proteins via the autophagy pathway, dismantling viral replication factories and inhibiting infection. This mechanism provides a theoretical foundation for marker-assisted breeding and the development of immunoenhancing agents or anti-disease biological products against GCRV-II infection. Future studies will focus on evaluating the protection efficacy of gcTrim47-based immunoenhancing agent against other pathogen infection and investigating evolutionary pressures driving the divergence of TRIM protein functions in teleost.

## Data Availability

The original contributions presented in the study are included in the article/**Supplementary Material**. Further inquiries can be directed to the corresponding author.

## Supplementary material

Supplementary Figure 1 The interaction and subcellular co-localization between gcTrim47/domain-deleted constructs and GCRV nonstructural proteins NS80/NS38 in 3-MA-treated, GCRV-infected CIK cells. **(A)** Interaction analyses between full-length gcTrim47 or its domain-deleted constructs and GCRV nonstructural proteins in CIK cells following co-treatment with 3-MA and GCRV infection. **(B, C)** Confocal microscopy analysis of subcellular co-localization between gcTrim47/domain-deleted constructs and GCRV nonstructural proteins NS38 **(B)** or NS80 **(C)** in 3-MA-treated, GCRV-infected CIK cells.
