# Supplementary figures and images for: Grass carp Trim47 restricts GCRV infection via SPRY domain-mediated autophagic degradation of nonstructural proteins and disruption of viral inclusion bodies
Source: Front Immunol. 2025 Jul 10;16:1623014. doi: 10.3389/fimmu.2025.1623014 (PMC12286827; doi:10.3389/fimmu.2025.1623014)

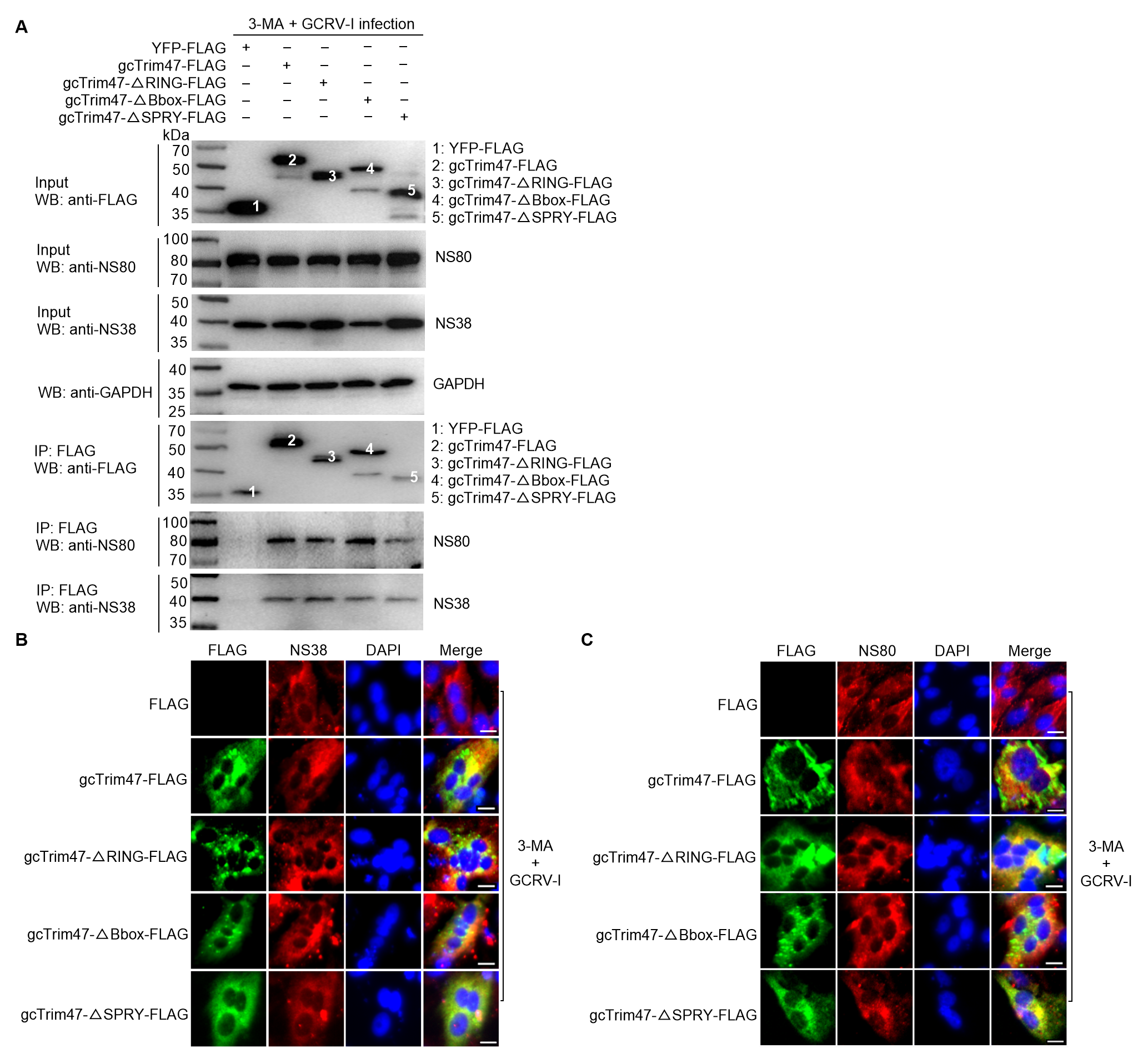

Supplement: Supplementary Figure 1 — The interaction and subcellular co-localization between gcTrim47/domain-deleted constructs and GCRV nonstructural proteins NS80/NS38 in 3-MA-treated, GCRV-infected CIK cells. (A) Interaction analyses between full-length gcTrim47 or its domain-deleted constructs and GCRV nonstructural proteins in CIK cells following co-treatment with 3-MA and GCRV infection. (B, C) Confocal microscopy analysis of subcellular co-localization between gcTrim47/domain-deleted constructs and GCRV nonstructural proteins NS38 (B) or NS80 (C) in 3-MA-treated, GCRV-infected CIK cells. [file Image1.tif]
